# Characterization of the ModABC Molybdate Transport System of *Pseudomonas putida* in Nicotine Degradation

**DOI:** 10.3389/fmicb.2018.03030

**Published:** 2018-12-10

**Authors:** Zhenyuan Xia, Liping Lei, Hong-Yue Zhang, Hai-Lei Wei

**Affiliations:** ^1^Key Laboratory of Microbial Resources Collection and Preservation, Ministry of Agriculture, Institute of Agricultural Resources and Regional Planning, Chinese Academy of Agricultural Sciences, Beijing, China; ^2^Yunnan Academy of Tobacco Agricultural Science, Kunming, China

**Keywords:** biodegradation, nicotine, *Pseudomonas putida*, molybdate transporter, ModABC

## Abstract

*Pseudomonas putida* J5 is an efficient nicotine-degrading bacterial strain that catabolizes nicotine through the pyrrolidine pathway. In our previous study, we used Tn5 transposon mutagenesis to investigate nicotine metabolism-associated genes, and 18 nicotine degradation-deficient mutants were isolated from 16,324 Tn*5*-transformants. Three of the mutants were Tn5 inserts into the *modABC* gene cluster that encoded an ABC-type, high-affinity, molybdate transporter. In-frame deletion of the *modABC* genes abolished the nicotine-degrading ability of strain J5, and complementation with *modABC* either from *P. putida* or *Arthrobacter oxidans* restored the degrading activity of the mutant to wild-type level. Nicotine degradation of J5 was inhibited markedly by addition of tungstate, a specific antagonist of molybdate. Molybdate at a non-physiologically high concentration (100 μM) fully restored nicotine-degrading activity and recovered growth of the *modABC* mutant in a nicotine minimal medium. Transcriptional analysis revealed that the expression of *modABC* was up-regulated at low molybdate concentrations and down-regulated at high moybdate concentrations, which indicated that at least one other system was able to transport molybdate, but with lower affinity. These results suggested that the molybdate transport system was essential to nicotine metabolism in *P. putida* J5.

## Introduction

Molybdenum (Mo) plays essential roles in bacteria, because it serves as a cofactor for a number of enzymes that catalyze a variety of oxidation/reduction reactions, and it is involved in microbial metabolism of carbon, nitrogen, and sulfur (Hille, 1996; Kisker et al., 1997). For the synthesis of molybdoenzymes, bacteria need to transport molybdate, activate it to an appropriate form, and incorporate it into the organic part of the molybdenum cofactor (Zhang et al., 2011). In nature, the predominant form of Mo is molybdate oxyanion, which is transported by an ABC-type transport system (Self et al., 2001). In *Escherichia coli*, the first identified Mo transporter was the high affinity ModABC transport system, in which ModA was responsible for molybdate binding, ModB was the transmembrane component of the permease, and ModC provided the energizer function on the cytoplasmic side of the membrane (Maupin-Furlow et al., 1995). Based on genome sequences, *mod* homologs can be identified in ≥50 bacteria (Leimkuhler and Iobbi-Nivol, 2015). Among these bacteria, *mod* genes have been functionally characterized in only a few species, which include *Rhodobacter capsulatus* (Wang et al., 1993), *Azotobacter vinelandii* (Luque et al., 1993), *Staphylococcus carnosus* (Neubauer et al., 1999), and *Bradyrhizobium japonicum* (Delgado et al., 2006). In *B. japonicum* USDA110, a nitrogen-fixing, root-nodule symbiont of soybean, *modA* and *modB* mutant strains were unable to grow with nitrate as a nitrogen source or as a respiratory substrate, and they lacked nitrate reductase activity (Delgado et al., 2006). Partially purified quinoline dehydrogenase from *P. putida* Chin IK indicated the presence of favin and molybdenum-binding pterin, and the enzyme activity was wholly dependent on the availability of molybdate in the growth medium (Blaschke et al., 1991).

Mo uptake occurred during nicotine biodegradation by gram-positive bacteria (Freudenberg et al., 1988; Menendez et al., 1997). The nicotine utilization of *Arthrobacter oxidans* P-34 (DSM419) required a supplementation with molybdate to the growth medium, and nicotine dehydrogenase (NDH) was identified as a molybdo-iron-sulfur-flavoprotein (Freudenberg et al., 1988). The nicotine-degrading plasmid pAO1 of *A. nicotinovorans* was sequenced and showed a remarkable gene cluster for biosynthesis of the molybdenum cytosine dinucleotide (MCD) cofactor and molybdate transporter system that were involved in nicotine degradation (Ganas et al., 2008). Despite the importance of Mo in nicotine metabolism by gram-positive bacteria, there has been very little work on the mechanisms involved in uptake of molybdate in nicotine metabolism by gram-negative bacteria. A recent report showed that conversion of 3-succinoylpyridine to 6-hydroxy-3-succinoylpyridine during the degradation of nicotine by *P. putida* S16 was catalyzed by a multi-enzyme reaction that consisted of a molybdopeterin binding oxidase (SpmA), molybdopterin dehydrogenase (SpmB), and a (2Fe-2S)-binding ferredoxin (SpmC) with molybdenum molybdopterin cytosine dinucleotide as a cofactor (Tang et al., 2013). This result led us to presume that Mo uptake was also involved in nicotine metabolism by gram-negative bacteria.

*Pseudomonas putida* J5 is an efficient nicotine-degrading strain isolated from the tobacco rhizosphere which could catabolize 3 g/l nicotine in 24 h (Wei et al., 2008). In our most recent study on nicotine-degrading associated genes in *P. putida* J5, we generated a Tn5 transposon mutant library with 16,324 transformants and screened 18 nicotinedegrading deficient mutants in which three mutants were mutated directly by Tn5 in the *modABC* transport gene cluster (Xia et al., 2015). In this paper, we cloned the entire gene cluster that encodes the molybdate transport system from strain J5, in-frame deleted the *modABC* genes, and characterized the function of the ModABC system in nicotine metabolism.

## Materials and Methods

### Strains, Plasmids, and Growth Conditions

Characteristics of strains and plasmids are listed in Table 1. *Pseudomonas putida* was grown in Luria-Bertani (LB) medium or NI medium at 28°C (Wei et al., 2009; Xia et al., 2015), and *Escherichia coli* was grown at 37°C in LB medium. For plasmid propagation and selection of transformants, media were supplemented with antibiotics at appropriate concentrations as follows: 100 μg/ml ampicillin, 50 μg/ml kanamycin, and 20 μg/ml tetracycline.

**Table 1:** Bacterial strains and plasmids used in this study.

Strain or plasmid	Genotype or relative phenotype	Source
**Escherichia coli**		
DH5α	F- recA1 endA1 hsdR17 supE44 thi-1 gyrA96 relA1Δ (argF-lacZYA)I169Φ80lacZ ΔM15	Sambrook and Russell, 1998
**Pseudomonas putida**		
J5	Apr; wild type	Wei et al., 2009
M430	Nicotine-degrading mutant inserted with Tn5	This study
M728	Nicotine-degrading mutant inserted with Tn5	This study
M9502	Nicotine-degrading mutant inserted with Tn5	This study
Δ*modABC*	*modABC* deletion mutant	This study
**Arthrobacter oxidans**		
J4	Nicotine-degrading bacterium	Lab collection
**Plasmids**		
pBluescript II SK+	ColE1 origin; Ap^r^	Stratagene
pHSG299	ColE1 origin; Km^r^	TaKaRa
pRK2013	ColE1 replicon with RK2 transfer region, helper plamsid; Km^r^	Figurski and Helinski, 1979
pRG970Km	Cloning vector containing promoterless *lacZYA* for construction of transcriptional fusions; Km^r^	Yan et al., 2009
pME6032	Shuttle vector; Tc^r^	Heeb et al., 2000
pBBR1MCS-2	Shuttle vector; Km^r^	Kovach et al., 1995
pHSG299Δmod	pHSG299::*modABC*	This study
p6032-modABC-J5	pME6032 with full length of *modABC* genes from J5	This study
p6032-modABC-J4	pME6032 with full length of *modABC* genes from J4	This study
pMCS2-modABC-J5	pBBR1MCS-2 with full length of *modABC* genes from J5	This study
pMCS2-modABC-J4	pBBR1MCS-2 with full length of *modABC* genes from J4	This study
p970-P*modABC*	pRG970Km with a 580 bp fragment containing the promoter of *modABC* genes	This study

### DNA Manipulations and Sequencing

Chromosomal and plasmid DNA isolations, restriction enzyme digestions, agarose gel electrophoresis, ligations, and *E. coli* transformations were performed according to standard protocols (Sambrook and Russell, 1998). Nucleotide sequencing was performed by Invitrogen Co., Ltd., China.

### Southern Hybridization and Cloning of the Tn5 Insertion Sites

To determine the copy number of the Tn5 transposon in mutant strains, total DNA was digested with *Eco*RI, *Pst*I, and *Bam*HI separated by electrophoresis on 0.8% (w/v) agarose gel and transferred onto nylon membranes (Hybond-N+; Amersham, GE Healthcare, Piscataway, NJ, United States). A digoxigenin-labeled, kanamycin-resistant, gene probe (Xia et al., 2015) was used to do hybridization and detection according to the protocol for the DIG High Primer DNA Labeling and Detection Starter Kit I (Roche).

Shotgun cloning was performed to determine the transposon insertion site. Chromosomal DNA samples were restricted with *Pst*I and *EcoR*I and ligated into pBluescript II SK. *E. coli* DH5α transformants were selected on LB medium that contained kanamycin. Positive clones were sequenced with Tn5-39 and Tn5-1571 (Xia et al., 2015) to allow determination of the precise location of a transposon insertion. Sequences were then compared to the protein sequence database (GenBank) using the BlastX algorithm (Altschul et al., 1990). For each mutant, the joins between the transposon sequences were identified.

### Construction of a *modABC* in-Frame Deletion Mutant

To create a *modABC* gene deletion allele, two fragments that flanked *modABC* genes were generated by endonuclease digestion. A 2.4 kb fragment was created by digestion with *Kpn*I and *Sal*I from pBS-M728, and another 1.6 kb fragment was generated by digestion with *Xho*I and *Eco*RI from pBS-M9502. After being digested with relevant restriction enzymes, the two fragments were inserted into pBluescript to create pBSΔmod. An approximately 4.0 kb long *Kpn*I-*Eco*RI fragment, which included the *modABC* genes with a 1.8 kb deletion, was lifted and ligated into pHSG299. The last suicide plasmid pHSG299Δmod was introduced into *P. putida* J5 by electroporation, and this was used in a two-step strategy to introduce the shortened *modABC* locus into the chromosome. Primers P3210 (5′-ACAGGTACCGCGCGCCTCTTC-3′) and P5864 (5′-AGTGGATCCCGGCAAAGTCGCTG) were used to confirm a double crossover event.

### Genetic Complementation of the *modABC* Mutant

To complement the *mod* mutant, a 2.6 kb fragment that contained the putative upstream promoter and coding region of the *modABC* genes was amplified from *P. putida* J5 with primers P3210 and P5864. Primers MJ4-F (5′-CT CAAGCTTGGGCAAGCGGCACTCG-3′) and MJ4-R (5′-CTCTCTAGAAGCGTGTCGCCATCGC-3′) were also used to amplify the *modABC* gene from *Arthrobacter oxidans* J4 to complement the *mod* mutant. The amplified intact *modABC* genes were inserted into the shuttle vector pME6032 or pBBR1MCS2, and the resulting plasmids were introduced into strain J5Δmod by triparental mating (Wei and Zhang, 2006).

### Transcription Analysis of *modABC* Genes

For determination of promoter activity, the promoter region that included the upstream fragment of *modABC* was amplified by PCR using primers P2925 (5′-GCGTGGGATCCATAATCGGA-3′) and P3505 (5′-CCATTCTGGATCCGCGCATA-3′), and a 580 bp *Bam*HI fragment was cloned ahead of a promoterless lacZ in p970Km (Yan et al., 2009), which is a derivative plasmid of pRG970 (Van den Eede et al., 1992). The resulting plasmid, p970-P*modABC*, was introduced into strain J5 and used to examine the activity of the *modABC* promoter. β-galactosidase measurement was performed at 6 hpi according to the standard protocol.

A further RT-qPCR assay was conducted to check the expression of *modABC*. Fresh midexponential cells from a single colony of strain J5 were harvested using 12,000 rpm centrifugation. Total RNA was extracted from the pellets using RNAprep pure cell/bacteria kit (Tiangen), treated with DNase, and reverse transcribed to cDNA using random hexamer primers and SuperScript III reverse transcriptase (Invitrogen). Transcriptional expression of *modA* was determined using the CFX Connect^TM^ Real-Time System (Bio-Rad) with Luna^®^Universal qPCR Master Mix (New England Biolabs). Transcript levels for *modA* were calculated relative to the level for the housekeeping gene *gyrA*.

### Detection of Nicotine Concentration

*P. putida* J5 and its derivatives were cultured to stationary phase in LB medium, and 1 ml of the cell suspension was spun down, inoculated into 100 ml of NI media that contained 1.0/l nicotine, and incubated at 30°C. One mM sodium tungstate and 1, 10, 100 μM, and 1 mM sodium molybdate were added as needed. To determine cell density, the absorbance (600 nm) of 3 ml of culture was determined with a spectrophotometer at 2 h intervals. The cell suspensions were then centrifuged, and the nicotine concentration of the supernatant was determined by high-pressure liquid chromatography (HPLC) (Wei et al., 2009).

## Results

### Isolation and Characterization of Nicotine-Degrading, Deficient Mutants M430, M728, and M9502

Previous analysis of the mutant strains M430, M728, and M9502 revealed that Tn5 was inserted into the homologs of *modB* and *modC* genes (Xia et al., 2015). Because the Tn5 insertion sites of M430 and M728 were very close to the *modB* gene, we only determined the degradation efficiency of mutants M728 and M9502, conducted Southern hybridization analysis, and subsequently cloned a larger fragment that covered the Tn5 transposon.

**FIGURE 1:**
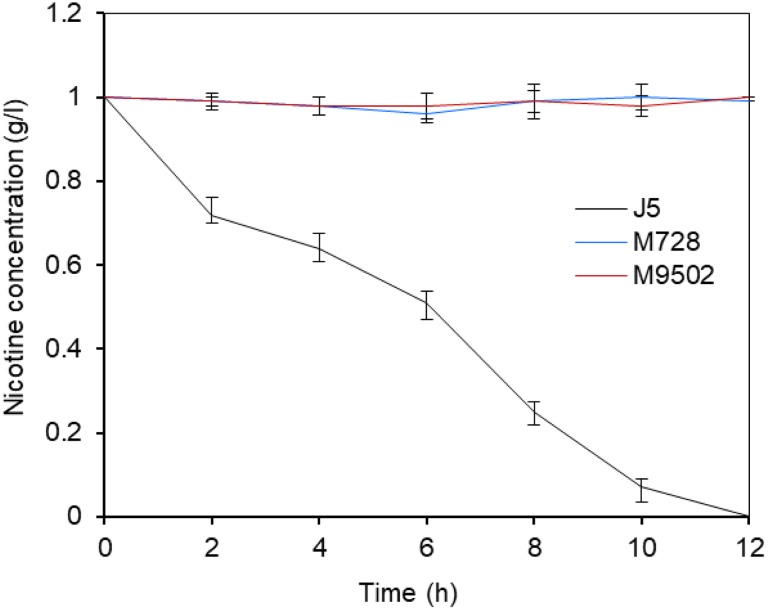
Nicotine degrading efficiency of *P. putida* J5 and the Tn5 insertion mutants M728 and M9502. All strains were grown in NI medium supplemented with 1 g/l nicotine and incubated at 30°C and 200 rpm. Nicotine degrading efficiency were monitored every 2 h according to a previous study (Wei et al., 2009). Presented data are average and standard error of the mean for at least three cultures which were assayed in duplicate.

**FIGURE 2:**
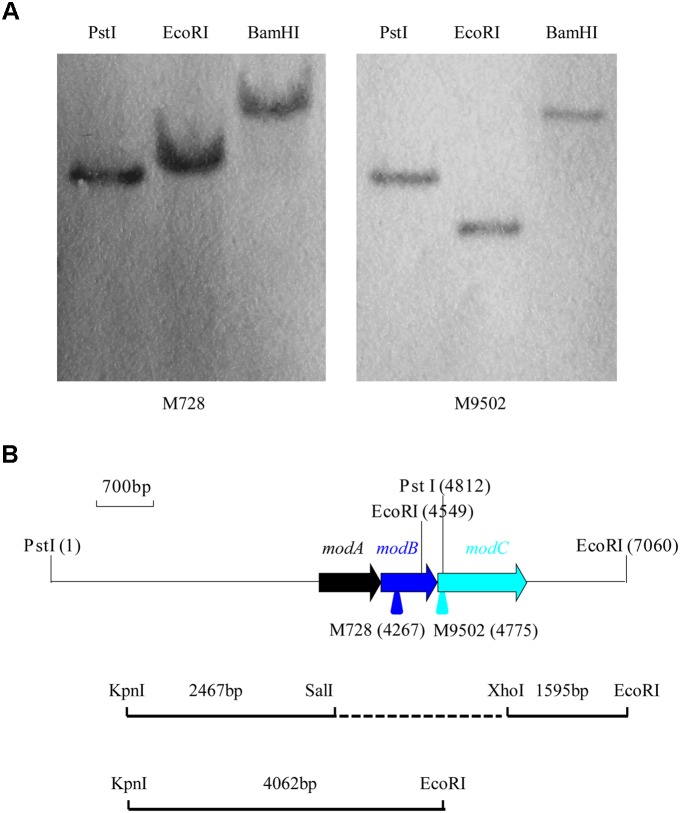
Southern hybridization of the nicotine-degrading deficient mutants M728 and M9502 and a diagram of the gene cluster of the *modABC* transport system and construction of the *modABC* deletion mutant. **(A)** Only one band was detected from the genomic DNA of M728 and M9502 after digestion with *Pst*I, *Eco*RI, and *Bam*HI. **(B)** Arrows represent the location and orientation of modABC genes. Suicide plasmid pHSG299 that contained the deleted *modABC* gene was constructed by ligating *Kpn*I- *Sal*I and *Xho*I- *EcoR*I fragments.

Under optimal conditions, wild type *P. putida* strain J5 thoroughly degraded 1.0 g/l nicotine in 12 h (Wei et al., 2009), but the Tn5 mutants, M728 and M9502, completely failed to degrade nicotine (Figure 1). The hybridization patterns of the two mutants were different (Figure 2A), which indicated that the mutants were not identical. Genomic sequences that flanked the insertion sites of M728 and M9502 were cloned by the shot-gun strategy with *Pst*I and *Eco*RI, based on the results of Southern blotting. The resulting plasmids, pBSM728-P and pBSM9502-E, contained about 6.3 and 3.6 kb foreign fragments, respectively, which included 1.6 kb of the mini-Tn5 sequence. After assembling the two fragments and discarding the Tn5 sequence, a 7.0 kb fragment was obtained (Figure 2B).

### *modABC* Genes

In the nucleotide sequence of the 7.0 kb fragment that was “rescued” from M728 and M9502, three open reading frames (ORFs) were found on one strand (Figure 2B). The genomic organization of the three ORFs suggested a transcriptional coupling. Potential Shine-Dalgarno sequences were identified at appropriate distances in front of the predicted start codons. These ORFs encoded 252 (26.7 kDa), 229 (24.2 kDa), and 363 (39.4 kDa) amino acid residues, which exhibited 88, 93, and 86% identities with ModA, ModB, and ModC in *P. putida* S16, respectively, which is a nicotine-degrading bacterium that has had the entire genome sequenced (Yu et al., 2011; Tang et al., 2013). Sequence analysis revealed accurately that the Tn5 cassettes of M728 and M9502 were inserted at the sites of 219 bp of *modB* and 36 bp of *modC*. The deduced *N*-terminus of ModA showed the typical features of lipoproteins (Sutcliffe and Russell, 1995). It resembled the molybdate-specific, periplasmic binding proteins. ModB of strain J5 was similar to the integral-membrane, channel-forming proteins of molybdate-specific, ABC transporters. ModC was predicted to be a cytosolic protein with typical consensus sequences for nucleotide binding. Additional studies focused on the function of ModABC in Mo uptake and nicotine biodegradation.

### Effect of *modABC* Mutation on Nicotine-Degrading Activity

To assess the function of the *modABC* genes with respect to the nicotine-degrading activity of *P. putida* J5, the *modABC* genes were also mutated by in-frame deletions in addition to the *mod*::Tn5 mutation (Figure 2B). The deletion-mutant was confirmed by PCR with primers P3210 and P5864. A 2.6 kb fragment was amplified from wild type strain J5, but only a 0.8 kb fragment was amplified from strain J5Δ*mod*, which indicated that a sequence of the *modABC* gene that contained approximately 1.8 kp had been deleted from strain J5 as designed.

**FIGURE 3:**
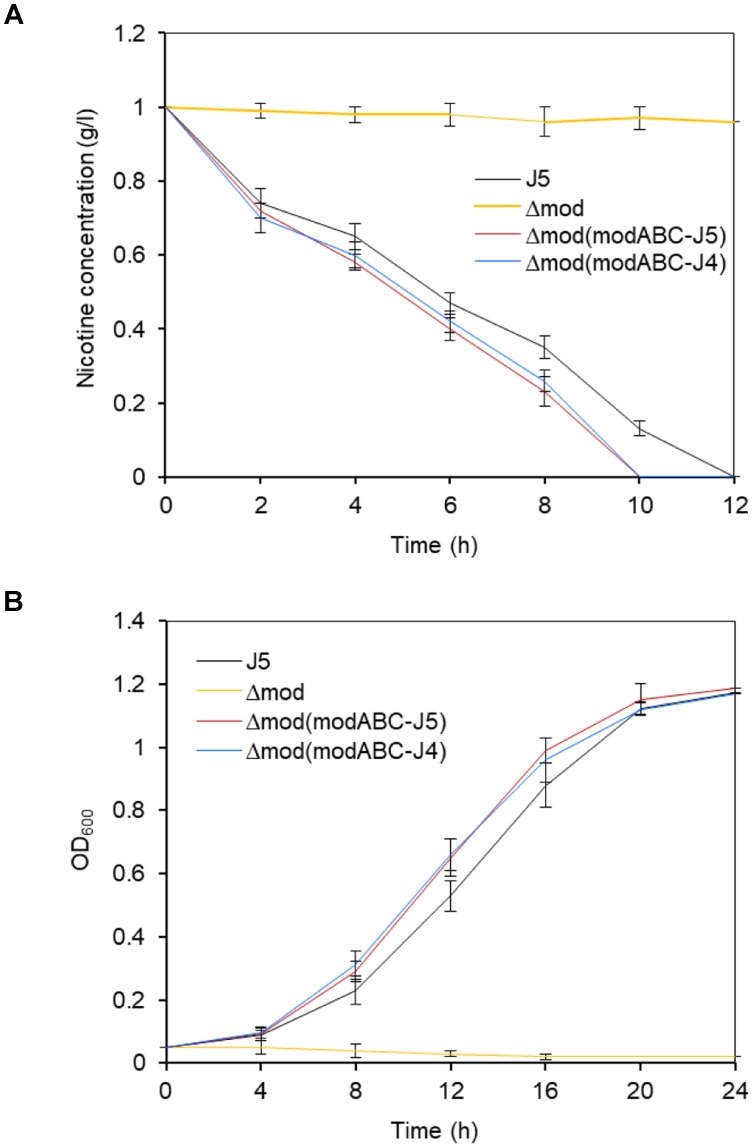
Time course of nicotine biodegradation **(A)** and growth **(B)** of *P. putida* J5 and the *modABC* mutants. All strains were grown in NI medium supplemented with 1 g/l nicotine and incubated at 30°C and 200 rpm. Culture samples were removed periodically to be measured for absorbance at 600 nm and the residual nicotine with HPLC. Presented data are averages and standard errors of the mean for at least three cultures that were assayed in duplicate.

In contrast to wild type *P. putida* J5, cells of the *modABC* mutant strain were unable to grow in a minimal medium with nicotine as the sole source of carbon and nitrogen (Figure 3B) but had a similar growth rate to the wild in the medium with glucose (Supplementary Figure S1). After incubating the cells under these conditions, almost no nicotine-degrading activity in cells of the *modABC* mutant was detected compared with those in cells of strain *P. putida* J5 (Figure 3A). Complementation of *P. putida* J5Δ*mod* with pME6032 that contained wild-type *modABC* genes of strain J5 restored both nicotine-degrading activity and the ability of the cells to grow in minimal medium with nicotine (Figures 3A,B). Similar results were obtained from complementation of *Δmod* with *modABC* genes of *A. oxidans* J4 (Figures 3A,B), which is an efficient nicotine-degrading, gram-positive bacterium that was isolated from tobacco rhizospheres. These results indicated that the molybdate transport system was involved in nicotine metabolism in *P. putida* J5.

### Effect of Molybdate on Nicotine Metabolism by *P. putida* J5 and the *modABC* Mutant

**FIGURE 4:**
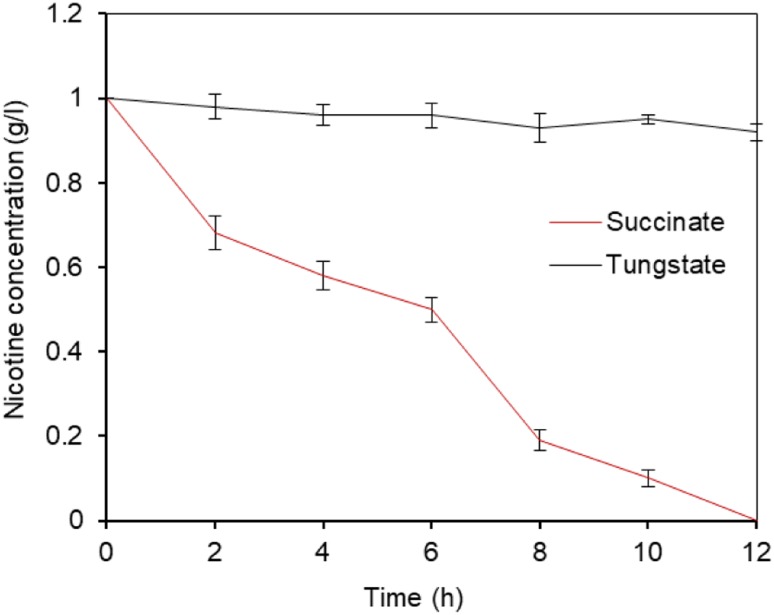
Nicotine degradation of *P. putida* J5 was inhibited by tungstate. *P. putida* J5 was cultured in NI medium with or without 1 mM sodium tungstate at 30°C and 200 rpm. Nicotine-degrading efficiency was monitored every 2 h as described above. Presented data are averages and standard errors of the mean for at least three cultures that were assayed in duplicate.

We tested to determine whether the growth and nicotine-degrading activity of wild type *P. putida* J5 was dependent on molybdate. Therefore, we employed tungstate, the specific antagonist of molybdate (Higgins et al., 1956; Nagel and Andreesen, 1989), to remove the molybdenum trace from the medium. We firstly determined if tungstate had impact on bacterial growth and nicotine metabolism. As expected, no difference was found on growth and metabolism when supplying 1 mM tungstate in LB liquid media with 1 g/l nicotine (Supplementary Figure S2). If the nicotine minimal growth medium was supplemented with tungstate (1 mM), no growth or nicotine consumption was found. These results seemed to be specific for nicotine, because no inhibition of growth and nicotine-degrading ability on succinate as a carbon source was detected after adding the same concentration of tungstate (Figure 4). Taken together, the results indicated that molybdate was a key element required by *P. putida* to degrade nicotine.

**FIGURE 5:**
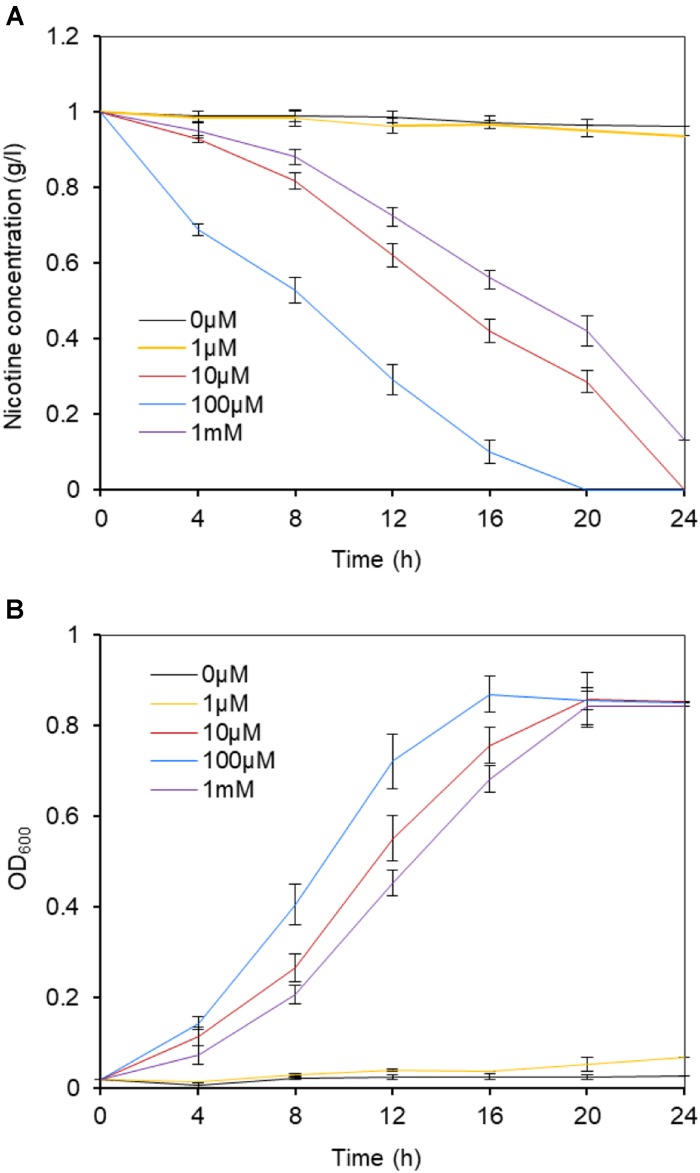
Nicotine degradation **(A)** and growth **(B)** of the *P. putida* J5 *modABC* mutant at a range of molybdate concentrations. The *modABC* mutant was cultured in NI medium supplemented with 1, 10, 100 μM, and 1 mM sodium molybdate. Culture samples were removed periodically to be measured for absorbance at 600 nm and the residual nicotine with HPLC. Presented data are averages and standard errors of the mean for at least three cultures that were assayed in duplicate.

Although we already proved ModABC constituted a molybdate transport system and played an important role in nicotine biodegradation in *P. putida* J5, we were not sure if increased concentrations of molybdate complemented the mutation of *modABC* mutants. Therefore, *P. putida* J5Δ*mod* was grown in minimal medium supplemented with nicotine and with different concentrations of molybdate. In the presence of molybdate, J5Δ*mod* revealed divergent kinetics of nicotine reduction and growth rate (Figure 5). The nicotine-degrading activity of the *P. putida* J5Δ*mod* was not influenced by supplementation with a low concentration of molybdate (1 μM). However, as the molybdate supplementation was increased, *P. putida* J5Δmod restored nicotine-degrading activity and recovered growth in the nicotine medium. At a concentration of 100 μM, molybdate had the highest efficiency in supporting the growth and nicotine-degrading activity of the *modABC* mutant, while a higher concentration of molybdate up to 1 mM repressed uptake of Mo and resulted in a decreased rate of growth and nicotine-degrading activity. The results clearly indicated that at least one other system was able to transport molybdate, but with lower affinity.

### Transcription of *modABC* Is Molybdate-Dependent

**FIGURE 6:**
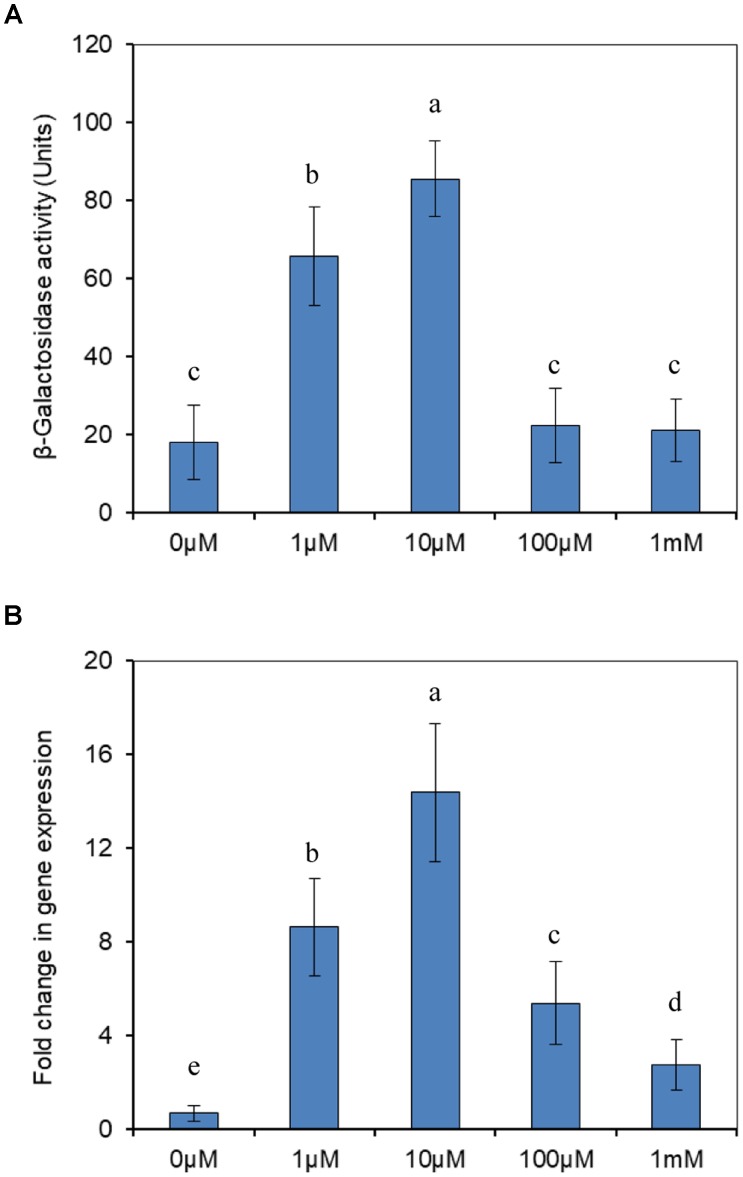
Transcriptional analysis of *modABC* genes. **(A)** Expression of β-galactosidase from a P*modABC*::*lacZ* fusion in *P. putida* J5. Cells were grown aerobically in NI medium supplemented with different concentrations of molybdate. **(B)** RT-qPCR analysis of the *modA* gene transcript produced in *P. putida* J5 that was grown as described above. Results presented in these histograms are the means of three independent experiments, and error bars indicate the standard deviations. Different letters represent significant differences between the treatments.

To investigate whether the expression of *modABC* genes was associated with molybdate, the effect of molybdate on the expression of *modABC* was first measured using a P*modABC*::*lacZ* transcriptional fusion in the plasmid p970Km. Before doing this assay, we firstly tested the growth of the wild type strain J5 supplied with selected titers of molybdate. No significant differences of the growth rates were found between the treatments (data not shown), which dismissed the possibility of growth affecting gene expression. The results revealed that *modABC* transcription under low-molybdate conditions was significantly enhanced compared with that under high-molybdate conditions, and 10 μM of molybdate induced the highest expression of *modABC*. After the molybdate concentration was increased to 100 μM, expression of the *modABC* genes were completely repressed (Figure 6A). We also employed RT-qPCR to double-check the expression of *modA* at different molybdate concentrations. The results were consistent with those in the promoter assay. The only exception was that *modABC* was induced moderately at higher molybdate concentrations (Figure 6B), which suggested that qPCR was more sensitive than transcriptional fusion. The expression of *modABC* was induced by the depletion of molybdate in *P. putida* J5, and the *modABC* molybdate transport system was a high affinity system.

## Discussion

*Pseudomonas putida* J5 is an efficient nicotine-degrading bacterium, which has been used to degrade employed in nicotine-contained tobacco waste (Wei et al., 2008). The molecular mechanisms of nicotine degradation by *P. putida* J5 was determined extensively by a genome-wide Tn5 mutagenesis strategy (Xia et al., 2015). In this study, we report on the physiological and genetic analysis of the three Tn5 mutants defective in *modABC* that encoded the molybdenum transport system. Both the random and site-directed mutants failed to degrade nicotine in minimal medium with nicotine as the sole source of carbon and nitrogen. Complementation of the mutants with native *modABC* genes or the homologs from well-studied *A. oxidans* fully restored nicotine-degrading activity. The effect of molybdate was observed for growth and utilization of the substrate of nicotine, as evidenced by the strong inhibition of tungstate, which was not observed for succinate, a control substrate reported before. The same phenomenon was also reported for degradation of quinolone and 2-furoic acid by *Pseudomonas* strains (Koenig and Andreesen, 1989; Blaschke et al., 1991). Overall, it seems likely that degradation by *N*-heterocyclic compounds generally involves a molybdenum-dependent reaction.

The trace element molybdenum is an important catalytic component of many enzymes involved in microbial metabolism of nitrogen, sulfur, and carbon. Enzymes that contain Mo at their active sites catalyze oxo-transfer reactions (Zhang et al., 2011). A variety of molybdoenzymes, such as xanthine oxidase, catalyze oxidative hydroxylation of a wide range of aldehydes and aromatic heterocycles (Stephan et al., 1996; Parschat et al., 2003; Wagener et al., 2009; Yokoyama and Leimkuhler, 2015). In the best-elucidated metabolic pathway of nicotine degradation by *A. nicotinovorans*, the heterotrimeric molybdenum enzyme NDH catalyzed the first nicotine-degrading reaction, which was hydroxylation of the pyridine ring to 6-hydroxynicotine (Brandsch, 2006; Fitzpatrick, 2018). But unfortunately, the nicotine oxidase NdaA from *P. putida* J5 and its homologs Nox from *Pseudomonas* sp. HZN6 and NicA2 from *P. putida* S16 have not shown such characters to known molybdoenzymes (Qiu et al., 2013; Tang et al., 2013). However, the key step in nicotine metabolism in the conversion of 3-succinoylpyridine to 6-hydroxy-3-succinoylpyridine by *P. putida* S16 was catalyzed by a multi-enzyme reaction that consisted of a molybdopeterin-binding oxidase (SpmA), molybdopterin dehydrogenase (SpmB), and a (2Fe-2S)-binding ferredoxin (spmC) with molybdenum molybdopterin cytosine dinucleotide as a cofactor (Tang et al., 2013). SpmABC proteins showed significant sequence identities to the subunits of the xanthine dehydrogenase family, such as NDH, a molybdoenzyme of *A. nicotinovorans* (Brandsch, 2006; Tang et al., 2013). The SpmABC homologs were also found from the draft genome of strain J5, which locates downstream of NadD, a homolog of Sapd in strain S16 (Tang et al., 2013). These reports support our conclusion of molybdenum-dependent nicotine degradation by *Pseudomonas* strains, although the SpmABC products await further purification and characterization.

It is very interesting to find that the ModABC transport system was repressed by a high concentration of molybdate, but 100 μM molybdate restored the growth and nicotine-degrading activity of the *modABC* mutant. In *Staphylococcus carnosus*, the mutant of *modABC* reduced nitrates very slowly in the absence of molybdate and efficiently in the presence of added molybdate (100 μM) (Neubauer et al., 1999). In *E. coli*, the molybdate transport system was repressed by increasing the molybdate concentration in the medium, and the *modABC* mutant utilized other transport systems with a lower affinity for molybdate (Rosentel et al., 1995). Molybdate can be taken up by the sulfate-transport system in the absence of a functional, high-affinity, molybdate-transport system in *E. coli* and *B. japonicum* (Rosentel et al., 1995; Delgado et al., 2006).

Our results, together with the similarity to previous reports, clearly indicated an involvement of the ModABC of *P. putida* J5 in molybdate transport. This is the first research to report that molybdenum and the ModABC transport system were involved in nicotine degradation by *P. putida*, but further research needs to be done to elucidate the other nicotine-degrading related ModABC transport systems.

## Author Contributions

H-LW and ZX designed the research. ZX, LL, and H-YZ performed the research. H-YZ and H-LW analyzed the data. H-LW wrote the paper.

## Conflict of Interest Statement

The authors declare that the research was conducted in the absence of any commercial or financial relationships that could be construed as a potential conflict of interest.

## Supplementary Material

FIGURE S1 Time course of growth of *P. putida* J5 and the *modABC* mutants in M9 medium with glucose as the sole source of carbon and nitrogen. All strains were incubated at 30°C and 200 rpm. Culture samples were removed periodically to be measured for absorbance at 600 nm. Presented data are averages and standard errors of the mean for at least three cultures that were assayed in duplicate.

FIGURE S2 Growth curve (up) and nicotine degradation of *P. putida* J5 in LB liquid media plus 1 g/l nicotine with or without 1 mM tungstate. All strains were incubated at 30°C and 200 rpm. Culture samples were removed periodically to be measured for absorbance at 600 nm. Presented data are averages and standard errors of the mean for at least three cultures that were assayed in duplicate.
