# Corrigendum: Characterization of the ModABC Molybdate Transport System of *Pseudomonas putida* in Nicotine Degradation

**DOI:** 10.3389/fmicb.2018.03213

**Published:** 2018-12-21

**Authors:** Zhenyuan Xia, Liping Lei, Hong-Yue Zhang, Hai-Lei Wei

**Affiliations:** ^1^Key Laboratory of Microbial Resources Collection and Preservation, Ministry of Agriculture, Institute of Agricultural Resources and Regional Planning, Chinese Academy of Agricultural Sciences, Beijing, China; ^2^Yunnan Academy of Tobacco Agricultural Science, Kunming, China

**Keywords:** biodegradation, nicotine, *Pseudomonas putida*, molybdate transporter, ModABC

In the published article, there was an error in affiliation one. Instead of “Yunnan Academy of Tobacco Agricultural Science, Kunming, China”, it should be “Key Laboratory of Microbial Resources Collection and Preservation, Ministry of Agriculture, Institute of Agricultural Resources and Regional Planning, Chinese Academy of Agricultural Sciences, Beijing, China.” In addition, there was an error regarding the affiliation for Zhenyuan Xia. As well as having affiliation one, they should also have “Key Laboratory of Microbial Resources Collection and Preservation, Ministry of Agriculture, Institute of Agricultural Resources and Regional Planning, Chinese Academy of Agricultural Sciences, Beijing, China.”

The authors apologize for this error and state that this does not change the scientific conclusions of the article in any way. The original article has been updated.

