# Supplementary figures and images for: Characterization of the ModABC Molybdate Transport System of Pseudomonas putida in Nicotine Degradation
Source: Front Microbiol. 2018 Dec 10;9:3030. doi: 10.3389/fmicb.2018.03030 (PMC6295455; doi:10.3389/fmicb.2018.03030)

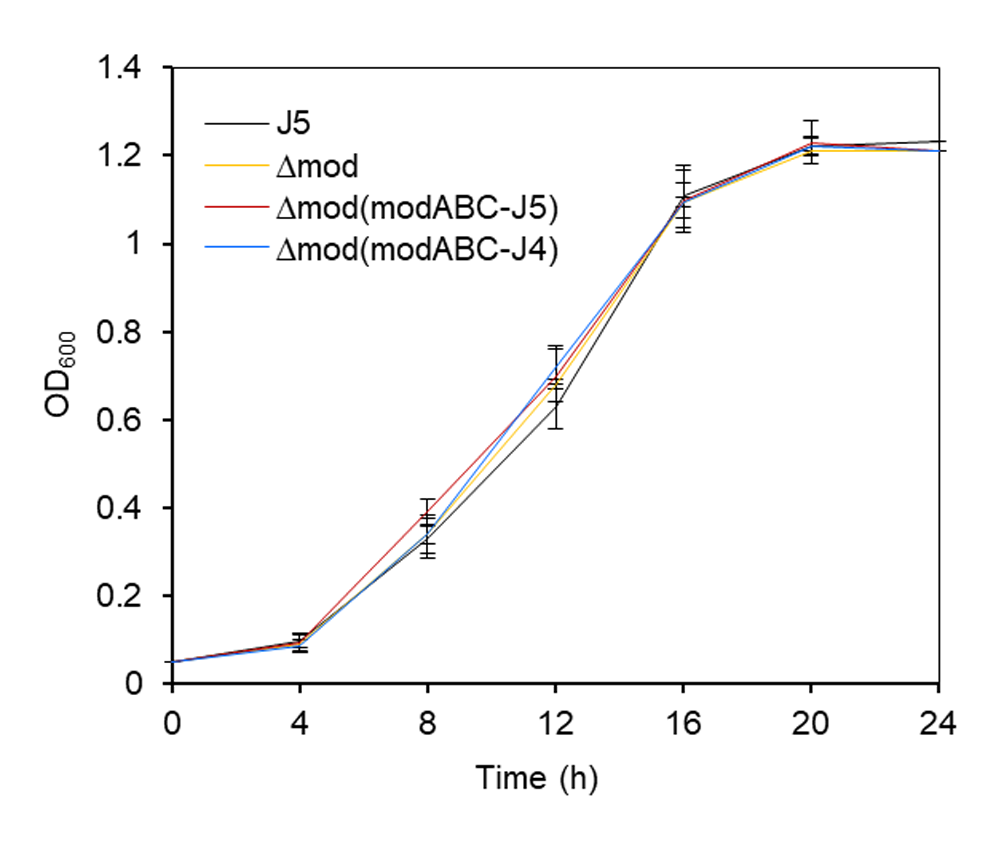

Supplement: FIGURE S1 — Time course of growth of P. putida J5 and the modABC mutants in M9 medium with glucose as the sole source of carbon and nitrogen. All strains were incubated at 30°C and 200 rpm. Culture samples were removed periodically to be measured for absorbance at 600 nm. Presented data are averages and standard errors of the mean for at least three cultures that were assayed in duplicate. [file Image_1.TIFF]

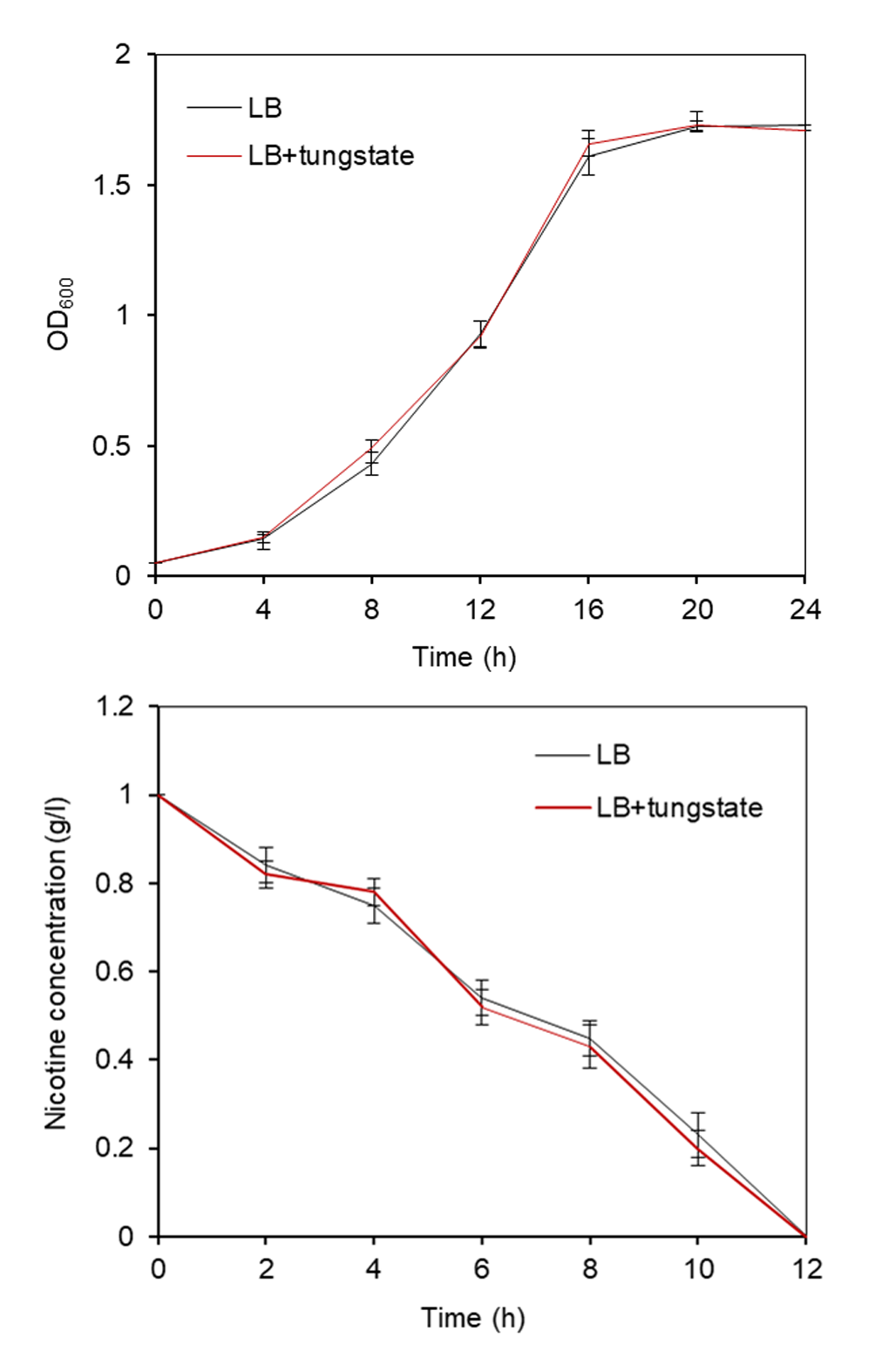

Supplement: FIGURE S2 — Growth curve (up) and nicotine degradation of P. putida J5 in LB liquid media plus 1 g/l nicotine with or without 1 mM tungstate. All strains were incubated at 30°C and 200 rpm. Culture samples were removed periodically to be measured for absorbance at 600 nm. Presented data are averages and standard errors of the mean for at least three cultures that were assayed in duplicate. [file Image_2.TIF]
